# 27229 Team Science: A Two-Year Follow-Up Case Study of Rutgers’ Ideation Forum

**DOI:** 10.1017/cts.2021.678

**Published:** 2021-03-30

**Authors:** Hosen Arman, Ziyad Razeq, Nancy Reichmann, Edmund Lattime, Biju Parekkadan

**Affiliations:** 1 Rutgers University Camden; 2 Rutgers Graduate School of Biomedical Science; 3 Rutgers Robert Wood Johnson Medical School; 4 Rutgers Cancer Institute of New Jersey; 5 Rutgers School of Engineering

## Abstract

ABSTRACT IMPACT: This study will provide important insight about effective team formation from coming up with an idea to successfully implementing that idea, as well as will highlight the implementation, evolution, and future directions of a team science initiatives. OBJECTIVES/GOALS: The goal of this study is to describe the feasibility of initiating an ideation forum to catalyze team formation, explore the process by which themes and teams are selected to participate in the forum setting, and assess the progress of participating teams post-forum through internal and external funding and other synergistic research activities. METHODS/STUDY POPULATION: Three ideation forums took place between 2018-2019 at Rutgers University, with a defined process and collection of data. The method of intervention to trigger team science, specifically the methodology employed to identify teams and produce new collaborative ideas, will first be described to show the feasibility of such an event to encourage team formation. In post-hoc analysis, we compare various success matrices of participating teams received seed funding versus teams that didn’t receive any funding to assess the progress of teams in the research ideation forum incubation process. RESULTS/ANTICIPATED RESULTS: Triggering team science through ideation forums is feasible and, in fact, quite productive to creating a durable response in formed teams showing continued productivity in publications, fundraising, and other academic metrics. DISCUSSION/SIGNIFICANCE OF FINDINGS: Our case review can illuminate how academic institutions can support team science research through ideation forums. In addition, this study lays an initial foundation for improvements in ideation forum creation and new metrics that can be shared broadly to compare across other institutions.

